# Apigetrin Promotes TNFα-Induced Apoptosis, Necroptosis, G2/M Phase Cell Cycle Arrest, and ROS Generation through Inhibition of NF-κB Pathway in Hep3B Liver Cancer Cells

**DOI:** 10.3390/cells11172734

**Published:** 2022-09-01

**Authors:** Pritam Bhagwan Bhosale, Abuyaseer Abusaliya, Hun Hwan Kim, Sang Eun Ha, Min Yeong Park, Se Hyo Jeong, Preethi Vetrivel, Jeong Doo Heo, Jin-A Kim, Chung kil Won, Hyun-Wook Kim, Gon Sup Kim

**Affiliations:** 1 Department of Veterinary Medicine, Research Institute of Life Science, Gyeongsang National University, Jinju 52828, Korea; 2 Biological Resources Research Group, Bioenvironmental Science & Toxicology Division, Korea Institute of Toxicology (KIT), 17 Jeigok-gil, Jinju 52834, Korea; 3 Department of Pharmacy, National University of Singapore, Singapore 117643, Singapore; 4 Department of Physical Therapy, International University of Korea, Jinju 52833, Korea; 5 Division of Animal Bioscience & Intergrated Biotechnology, Jinju 52725, Korea

**Keywords:** flavonoids, apigetrin, reactive oxygen species, necroptosis, apoptosis, liver cancer

## Abstract

Apigetrin (7-(β-D-glucopyranosyloxy)-4′,5-dihydroxyflavone), a glycoside bioactive dietary flavonoid derived from *Taraxacum officinale* and *Teucrium gnaphalodes,* is known to possess anticancer, antioxidant, and anti-inflammatory effects on numerous cancers. In the present study, we examined the effect of apigetrin in Hep3B hepatocellular cancer cell line (HCC). Apigetrin inhibited cell growth and proliferation of Hep3B cells, as confirmed by MTT and colony formation assay. We used apigetrin at concentrations of 0, 50, and 100 µM for later experiments. Of these concentrations, 100 µM of apigetrin showed a significant effect on cell inhibition. In apigetrin-treated Hep3B cells, cell cycle arrest occurred at the G2/M phase. Apoptosis and necroptosis of Hep3B cells treated with apigetrin were confirmed by Annexin V/propidium iodide (PI) staining and flow cytometry results. Morphological observation through 4′,6-diamidino-2-phenylindole (DAPI) staining showed intense blue fluorescence representing chromatin condensation. Hematoxylin staining showed necroptotic features such as formation of vacuoles and swelling of organelles. Apigetrin increased reactive oxygen species (ROS) levels in cells, based on fluorescence imaging. Furthermore, the underlying mechanism involved in the apoptosis and necroptosis was elucidated through western blotting. Apigetrin up-regulated TNFα, but down-regulated phosphorylation of p-p65, and IκB. Apigetrin inhibited the expression of Bcl-xl but increased Bax levels. Up-regulation of cleaved PARP and cleaved caspase 3 confirmed the induction of apoptosis in apigetrin-treated Hep3B cells. Additionally, necroptosis markers RIP3, p-RIP3, and p-MLKL were significantly elevated by apigetrin dose-dependently, suggesting necroptotic cell death. Taken together, our findings strongly imply that apigetrin can induce apoptosis and necroptosis of Hep3B hepatocellular cancer cells. Thus, apigetrin as a natural compound might have potential for treating liver cancer.

## 1. Introduction

Hepatocellular cancer (HCC) is the fourth most prevalent malignancy worldwide. It is a global health concern with an expected incidence of more than 1 million cases by 2025 [1]. Infections with hepatitis B or C virus, cirrhosis, steatohepatitis, and underlying liver diseases contribute to the occurrence of hepatocellular cancer [2]. Appropriate staging of HCC can help us decide therapeutic treatment strategies [3]. Sorafenib is a documented medication that can improve overall survival of patients with advanced stages of HCC [4,5]. However, fewer than one-third of patients respond to Sorafenib treatment. In addition, resistance to Sorafenib can develop within six months. Long-term use of chemotherapy drugs can also lead to toxicity, gastrointestinal lesions, hair loss, and neurological dysfunction [5]. Chemotherapeutic drugs can also affect normal cells due to toxicity and metabolic alteration. Therefore, an effective treatment with fewer side effects is needed.

Programmed cell death (PCD) is the most investigated and comprehensively studied anticancer mechanism [6]. Apoptosis, necroptosis, and autophagy are the most common controlled cell death signaling processes involved in cancer cell death [7,8]. Apoptosis is a commonly regulated cell death mechanism in cancer cells, regulated by two common pathways depending on the stimuli-mitochondrial intrinsic and extrinsic death receptor pathways [9]. These apoptosis pathways can cause activation of caspase-3 and caspase-7, and further cleavage of PARP, leading to caspase-dependent nucleosome fragmentation [10]. TNFα is an inflammatory cytokine that can trigger a diverse range of signaling processes within cells, including apoptosis and necroptosis [11]. TNFα can activate receptor-interacting serine/threonine-protein kinase 1 (RIPK1) and cellular inhibitor of apoptosis protein 1 (cIAP1), eventually phosphorylating the inhibitor of kappa B kinase (IκB) complex and leading to nuclear factor kappa B (NF-κB) activation [12]. RIPK1 can form a complex with Fas-associated protein and RIPK3 in cells lacking caspase activity. The necroptotic pathway is initiated by oligomerizing mixed lineage kinase domain-like protein (MLKL) via activated RIPK3 [13].

Persistent high levels of ROS can damage DNA through strand breaks and base oxidation if not repaired, inducing apoptosis or necroptosis [14]. Plant-derived compounds can cause cancer cell death through ROS generation [15]. Disproportional ROS levels significantly induce apoptosis, necroptosis, cell inhibition, and cell cycle arrest [16].

Naturally occurring phytochemicals play an important role in the treatment and prevention of a variety of malignancies [17]. Flavonoids are essential therapeutic adjuvants for several diseases, including arteriosclerosis, diabetes, neurological disorders, and cancers [18]. Considerable experimental evidence has shown their anticancer potential in numerous cancer cell lines [15,18]. Anticancer effects of flavonoids can be evaluated by cell cycle arrests, apoptosis, autophagy, reactive oxygen species (ROS) modulation, suppression of cell invasion, and proliferation [19,20]. Almost all clinically used antitumor drugs have toxic side effects. However, it has been suggested that flavonoids could decrease toxic effects during chemotherapy due to their antioxidant potential [21,22]. Consumption of fruits and vegetables containing flavonoids has preventive roles against cancer and other diseases [23]. Apigetrin (apigenin 7-glucoside) have antibacterial, antifungal, and nonsteroidal anti-inflammatory properties [24]. Compound apigetrin has better solubility and stability than other flavonoids [25]. Apigetrin exhibits an apoptotic effect by inducing ROS generation and STAT3/JAK2 signaling pathway in gastric cancer cells [26]. Upon treatment with apigetrin, cervical cancer cells (HeLa) undergone cell apoptosis through the PTEN/PI3K/AKT pathway and inhibition of cell migration [27].

Despite a few studies have been conducted on the anticancer and chemo preventive effect of apigetrin, the mechanism involved in its effect on Hep3B cells remains elusive. Thus, the objective of the current study was to explore the anticancer potential of apigetrin in Hep3B cells and the molecular mechanism of action involved.

## 2. Materials and Methods

### 2.1. Cell Line and Chemicals

Hep3B human liver cell line obtained from Korea cell line (Seoul, Korea). Fetal bovine serum (FBS), antibiotics penicillin/streptomycin (P/S), phosphate-buffered saline (PBS), and Dulbecco’s modified Eagle’s medium (DMEM) were purchased from Gibco (BRL Life Technologies, Grand Island, NY, USA). Propidium iodide (PI) and 3-(4,5-Dimethylthiazol-2-yl)-2,5-diphenyltetrazolium bromide (MTT) were obtained from Sigma–Aldrich (St. Louis, MO, USA). DAPI (4′, 6-Diamidino-2-phenylindole) was purchased from Vector Laboratories Inc. (Burlingame, CA, USA). Apigetrin was obtained from InterPharm (KOYANG-SI, South Korea). Materials and chemicals used for electrophoresis were bought from Bio-rad (Hercules, CA, USA). Primary antibodies against Bcl-xl (cat. no. 2762S), Bax (cat. no. 2774S), Caspase 3 (cat. no. 96623), Cleaved caspase 3 (cat. no. 9664S), poly ADP-ribose polymerase (PARP) (cat. no. 9542S), cleaved-PARP (cat. no. 5625S), p-MLKL (cat. no. 91689S), p-RIP3 (cat. no. ab209384), MLKL (cat. no. 14993S), TNFα (cat. no. 3707S), p-p65 (cat. no. 3033S), p-IκB (cat. no. 2859S), P65 (cat. no. 8242S), IκB (cat. no. 4812S), and β-actin (cat. no. 3700S) were purchased from Cell Signaling Technology (Danvers, MA, USA). Horseradish peroxidase (HRP)-conjugated secondary antibodies were obtained from Bethyl Laboratories, Inc. (Montgomery, AL, USA).

### 2.2. Apigetrin Treatment and Cell Viability Assay

The viability of cells was assessed by MTT assay. Briefly, Hep3B cells were seeded into a 48-well plate at a density of 3 × 10^4^ cells per well and then treated with (0, 25, 50, 100, and 200 μM) of apigetrin or vehicle (dimethyl sulfoxide, DMSO) alone for 24 and 48 h. MTT was added to each well followed by incubation at 37 °C for 2 h and supernatants were carefully removed. Purple formazan crystals generated in each well were dissolved in 200 µL of DMSO. After 15 min of shaking, the absorbance of each well was measured at 540 nm using a PowerWave HT microplate spectrophotometer (BioTek, Winooski, VT, USA).

### 2.3. Colony Formation Assay

Hep3B cells were seeded into a 6-well plate at 1000 cells/well and cultured for 24 h. These cells were incubated with (0, 50, and 100 μM) apigetrin for about two weeks. The medium was changed for every three days. After the incubation period, colonies formed on the plate were fixed with 4% paraformaldehyde for 30 min, followed by staining with 0.6% Giemsa stain for 30 min. After excess stain was removed with running tap water, the number of colonies was counted with ImageJ software (U.S. National Institutes of Health, Bethesda, MD, USA).

### 2.4. DAPI Staining of Apoptotic Cells

Hep3B cells were seeded into a 12-well plate at a density of 1 × 10^4^ cells/well and treated with apigetrin (0, 50, and 100 μM) for 48 h. After incubation cells were rinsed with 1X PBS and then fixed with 4% paraformaldehyde at room temperature (RT) for 10 min. Cells were further stained with DAPI solution (10 µg/mL) for 15 min at RT and then analyzed with a fluorescence microscope (Nikon, ECLIPSE Ti-U).

### 2.5. Hematoxylin Staining for Observing Cell Death Morphology

Hep3B cells were seeded into 6-well plates at a density of 5 × 10^4^ cells/well and then treated with apigetrin (0, 50, and 100 μM) for 48 h. After 48 h treatment, cells were rinsed three times with 1X PBS and then fixed with 4% formaldehyde solution at RT. Following fixation, cells were washed with 1X PBS twice before they were stained with 500 µL of Mayer’s stain (Cancer Diagnostics, Inc., Durham, NC, USA). Stained cells were kept for 20–30 min at RT and then viewed under a microscope using 90% glycerol as a mounting solution.

### 2.6. Cell Cycle Analysis

Hep3B cells were seeded onto 60-mm plates at a density of 4 × 10^5^ cells/plate and treated with indicated concentrations (0, 50, and 100 μM) of apigetrin to analyze the effect on cell cycle phases concerning DNA contents. Cells were rinsed with ice-cold 1X PBS, trypsinized, and centrifuged. Cells were fixed with 70% ethanol for about 30 min at 4 °C. Fixed cells were then stained with PI (50 µg/mL) and RNaseA (0.1 mg/mL) at RT for 30 min. These cells were examined by flow cytometry using a Cytomics FC 500 (Beckman Coulter, Brea, CA, USA). For each sample, around 10,000 cells were sorted. Data collected were analyzed with a CXP Software (Beckman Coulter, Inc., Fullerton, CA, USA).

### 2.7. Annexin V-Staining for Cell Death Analysis

Cell death analysis of Hep3B cells treated with apigetrin was performed with an allophycocyanin (APC)/Annexin V apoptosis detection kit (BD Biosciences, San Diego, CA, USA) following the manufacturer’s protocol. Briefly, Hep3B cells were seeded onto 60-mm plates at a density of 4 × 10^5^ cells/plate and treated with indicated concentrations (0, 50, and 100 µM) of apigetrin for 48 h. After incubation, cells were collected and washed with 1X PBS, followed by centrifugation. Cells were resuspended in binding buffer for 20 min in the dark followed by staining with Annexin V and PI at RT. Flow cytometry analysis was performed after data were obtained with a FACS Calibur flow cytometer (BD Biosciences, San Jose, CA, USA).

### 2.8. Determination of Reactive Oxygen Species

ROS generation in apigetrin-treated cells was determined using molecular probes from Invitrogen detention technologies. Hep3B cells were seeded into 96-well plates at a density of 5 × 10^4^ cells/well and treated with apigetrin (100 µM), NAC (N-acetyl-l-cysteine, 10 mM), and H_2_O_2_ (100 µM). By dissolving fluorescein carboxy-H2DCFDA (C4000) in ethanol (100%), a molecular probe derivative was made. After adding 5 µL of the dye’s working solution to cells, the mixture was incubated at RT for no more than 15 min. The intensity produced was evaluated using a fluorescence absorbance spectrometer with an excitation/emission range of 492/520 nm. The relative quantity of ROS produced was then calculated.

### 2.9. Western Blot Analysis

Hep3B cells were seeded in 60-mm plates 5 × 10^5^ cells/plate and treated with apigetrin (0, 50, and 100 µM) for 48 h. After incubation, cells were lysed using RIPA (iNtRON Biotechnology, Seoul, South Korea) containing a protease inhibitor and a phosphatase inhibitors cocktail (Thermo Scientific, Rockford, IL, USA) and centrifuged at 10,000 rpm for 10 min at 4 °C. A PierceTM BCA (Thermo Fisher Scientific, Rockford, IL, USA) was used to determine the concentration of extracted protein. Proteins were separated with SDS polyacrylamide gel (8–15%) electrophoresis and then transferred onto polyvinylidene fluoride (PVDF) membranes (Immobilon-P, 0.45 mm; Millipore, Billerica, MA, USA) utilizing a semi-dry transfer technique (Atto Corp., Tokyo, Japan). These membranes were blocked with 5% bovine serum albumin (BSA) in Tris-buffered saline containing 1% Tween 20 (TBS-T, pH 7.4), 5% BSA (bovine serum albumin), or 1X phosphoblocking solution at RT for 1 h and then incubated overnight with primary antibody 1:1000 at 4 °C, then followed by 2 h of secondary antibody at RT. An electrochemiluminescence (ECL) detection device (Bio-Rad Laboratory, Hercules, CA, USA) was used to detect proteins. ImageJ software (U.S. National Institutes of Health, Bethesda, MD, USA) was used to quantify protein expression.

### 2.10. Statistical Analysis

All experimental results are provided as mean ± standard deviation (SD) of triplicate samples using GraphPad Prism tool. One-way analysis of variance (ANOVA) was used to quantify significant differences between two groups, followed by Dunnett’s test. * *p* < 0.05, ** *p* < 0.01, *** *p* < 0.001.

## 3. Results

### 3.1. Cytotoxic and Inhibitory Effect of Apigetrin on Hep3B Cells

**Figure 1:**
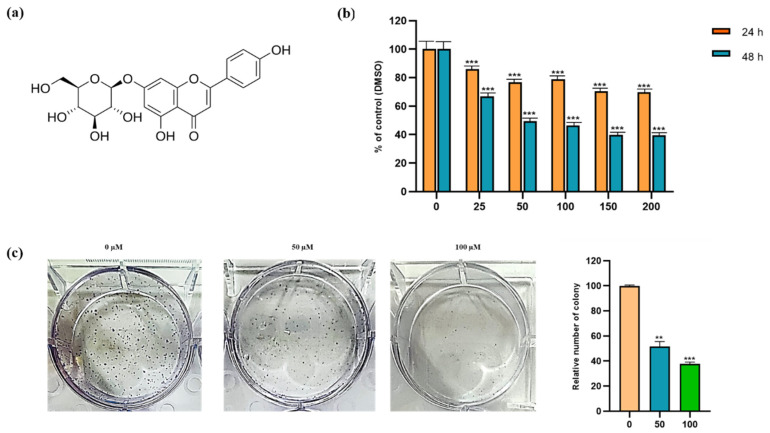
Cytotoxic and inhibitory effects of compound apigetrin. (**a**) Structure of apigetrin. (**b**) Hep3B cells were incubated with different concentrations (0, 25, 50, 100, 150, and 200 µM) of apigetrin for 24 and 48 h. The control group (0 μM) was treated with the same amount of DMSO. *** *p* < 0.001 vs. control. (**c**) Hep3B cells were treated with indicated concentrations (0, 50, and 100 µM) for 10 days and further stained with Giemsa solution to determine the survival percentage of colony formation in Hep3B cells after treatment with apigetrin. Results obtained from three independent experiments were expressed as mean ± standard deviation (SD) compared with the control group. ** *p* < 0.01 vs. control; *** *p* < 0.001 vs. control.

The cytotoxic and inhibitory effects of apigetrin on Hep3B cells were evaluated by MTT assay. After Hep3B cells were incubated with 0, 25, 50, 100, 150, and 200 µM of apigetrin for 24, and 48 h, results indicated that apigetrin reduced cell viability in a dose and time-dependent manner (Figure 1b). The half-maximal inhibitory concentration (IC50) of apigetrin was 52.67 µM at 48 h. In connection to this, significant cell death was observed after treatment with apigetrin at 50, and 100 µM for 48 h. A colony formation assay was used to determine effects of apigetrin on cell growth for a longer period. Apigetrin treatment significantly reduced the formation of colonies in cells compared to the control (Figure 1c). Thus, we investigated the mechanism involved in the cytotoxic effect of cell death in apigetrin-treated Hep3B cells.

### 3.2. Apigetrin Induces G2/M Phase Cell Cycle Arrest in Hep3B Cells

**Figure 2:**
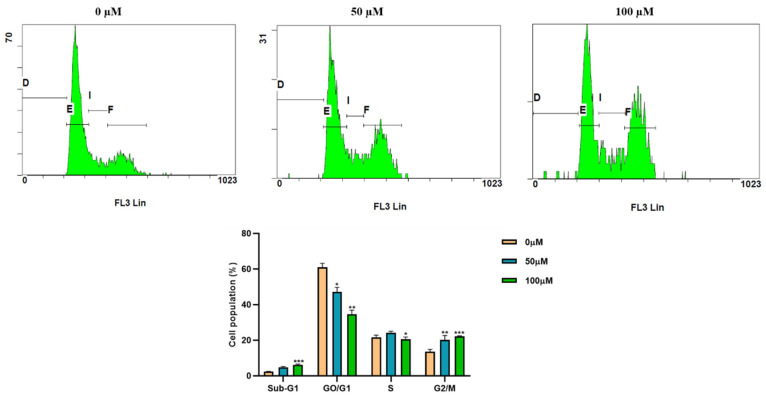
Cell cycle analysis by flow cytometry. Hep3B cells were treated with indicated concentrations (0, 50, and 100 µM) of apigetrin for 48 h, stained with PI, and cell cycle distribution was analyzed by flow cytometry. Values are given as mean ± standard deviation (SD) of three independent experiments. * *p* < 0.05 vs. control; ** *p* < 0.01 vs. control; *** *p* < 0.001 vs. control.

We performed cell cycle analysis to investigate the mechanism of cell growth inhibition using flow cytometry. Hep3B cells were treated with indicated concentrations (0, 50, and 100 µM) of apigetrin for 48 h. The number of cells in the G2/M phase was markedly increased in apigetrin-treated groups compared to the control group. Sub-G1 phase cell population was decreased at higher concentrations of apigetrin-treated Hep3B cells (Figure 2).

### 3.3. Apigetrin Induces Apoptotic and Necroptotic Cell Death in Hep3B Cells

**Figure 3:**
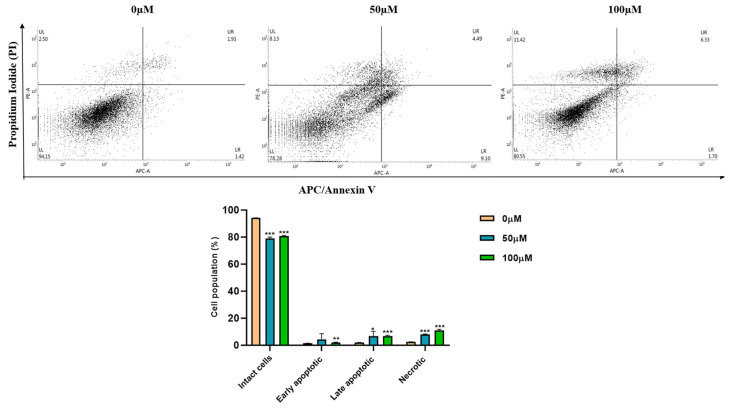
Double staining analysis by flow cytometry. Hep3B cells were treated with different concentrations (0, 50, and 100 µM) of apigetrin for 48 h. After allophycocyanin (APC)/Annexin V and propidium iodide (PI) double-staining was done cells were analyzed by flow cytometry. Values are given as mean ± standard deviation (SD) of three independent experiments. * *p* < 0.05 vs. control, ** *p* < 0.01 vs. control, *** *p* < 0.001 vs. control.

In apigetrin-treated Hep3B cells, apoptotic and necroptotic cell death populations were determined by double staining followed by flow cytometry. As shown in Figure 3, apigetrin treatment significantly increased the population of late apoptotic (right upper quadrant) and necroptotic (upper left quadrant) cells. These results indicate that apigetrin can induce apoptotic and necroptotic cell death in Hep3B cells.

### 3.4. Apigetrin Causes Morphological and Nuclear Alterations of Hep3B Cells

**Figure 4:**
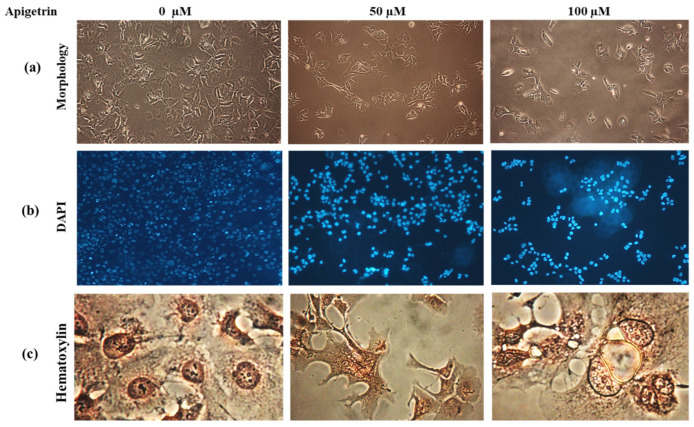
Morphological and nuclear changes upon apigetrin treatment. (**a**) Changes in the morphology of Hep3B cells after 48 h of apigetrin treatment under a light microscope, showing cell death after treatment with apigetrin at 50, and 100 µM. (**b**) After treatment with apigetrin (0, 50, and 100 µM) for 48 h, DAPI staining was performed followed by observation under a fluorescence microscope. (**c**) Hematoxylin staining was performed for cells after treatment with apigetrin at different concentrations (0, 50, and 100 µM) for 48 h. Hep3B cells showed necroptotic morphology such as cell swelling and formation of vacuoles.

Upon apigetrin treatment for 48 h, the morphology of Hep3B cells differed from that of control cells. Under a light microscope, morphological changes such as cell shrinkage, decreased cell count, and floating cells were observed as shown in Figure 4a. Nuclear changes were observed after DAPI staining where a bright blue fluorescence representing chromatin pyknosis and condensation was observed after treatment with apigetrin (Figure 4b). Similarly, hematoxylin staining of cells treated with apigetrin showed morphological features of vacuole formation and swelling of organelles indicating necroptosis (Figure 4c). These morphological features confirmed that apigetrin caused changes in nuclear morphology of Hep3B cells.

### 3.5. Apigetrin Increases the Generation of ROS in Hep3B Cells

**Figure 5:**
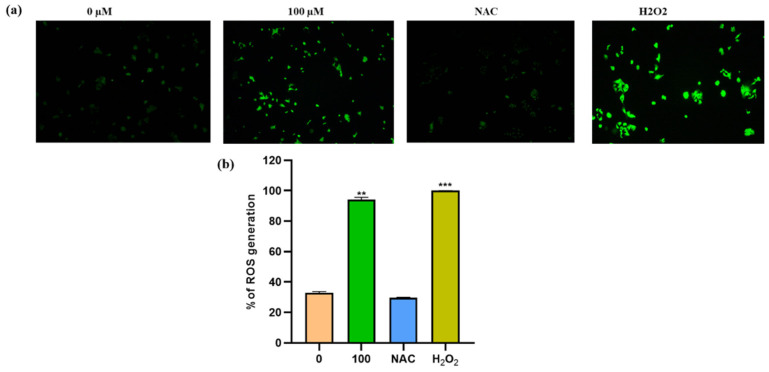
Measurement of ROS levels in Hep3B cells. (**a**) Changes in ROS production were detected by DCFDA fluorescence intensity using a fluorescence microscope. (**b**) Levels of ROS generation were measured by fluorescent intensity detection in apigetrin-treated Hep3B cells. Values are given as mean ± standard deviation (SD) of three independent experiments. ** *p* < 0.01 vs. control, *** *p* < 0.001 vs. control.

ROS production plays an important role in the death of cancer cells. ROS levels were measured using spectroscopic fluorescence and microscopic observations to see if apigetrin caused cell death by inducing ROS in Hep3B cells. Results showed that apigetrin caused ROS production as measured by spectroscopic fluorescence. NAC pretreatment for 1 h decreased ROS production in cells. However, cells treated with apigetrin showed increased ROS production. Cells treated with H_2_O_2_ were used as a positive control. NAC treatment attenuated the production of ROS as evidenced by fluorescence microscopy and spectroscopic fluorescence analysis (Figure 5a). Data from microscopic examination and spectroscopic fluorescence analysis suggest that apigetrin can induce ROS production in cells. (Figure 5b).

### 3.6. Effect of Apigetrin on Inflammatory Pathway in Hep3B Cells

**Figure 6:**
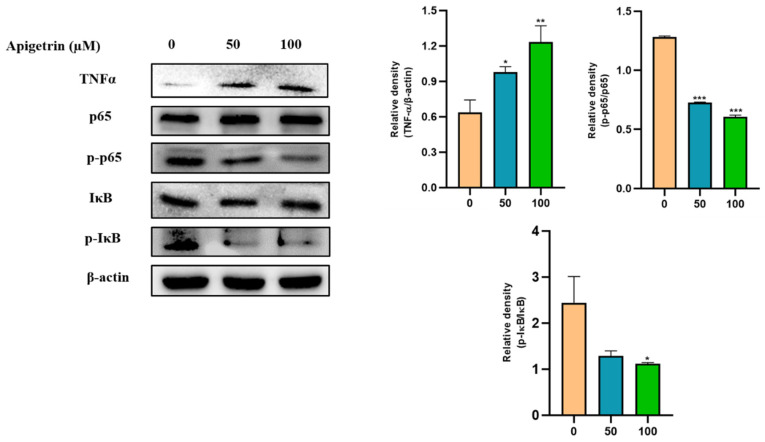
Effects of apigetrin on inflammatory markers in Hep3B cells. Proteins were isolated from cells, and expression levels of inflammatory marker proteins TNFα, p-p65, and p-IκB were analyzed by western blot. Expression levels are presented as a mean ± standard deviation (SD) graphically based on their densitometry values from with three independent experiments. * *p* < 0.05 vs. control, ** *p* < 0.01 vs. control, *** *p* < 0.001 vs. control.

To better understand the molecular mechanism involved in the effect of apigetrin on Hep3B cells, inflammatory markers were analyzed. Western blot analysis showed that TNFα expression was increased by apigetrin dose-dependently. Expression levels of NF-κB pathways proteins were also investigated through immunoblot assay. Results showed that expressions levels of p-P65 and IκB levels were decreased in apigetrin-treated cells compared to those in control cells (Figure 6).

### 3.7. Effects of Apigetrin on Apoptotic Proteins in Treated on Hep3B Cells

**Figure 7:**
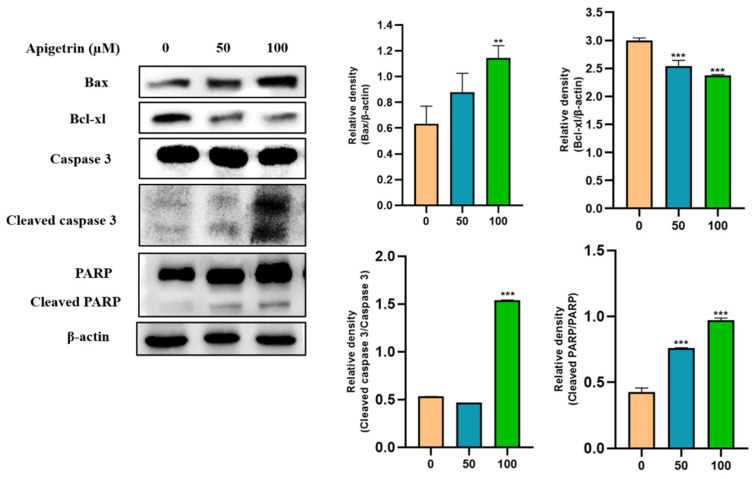
Immunoblot analysis of apoptotic proteins in Hep3B cells after treatment with apigetrin. Apigetrin was used to treat cells at indicated concentrations. Cell lysates were subjected to western blot analysis using Bax, Bcl-xl, cleaved caspase 3 and cleaved PARP primary antibodies. Expression levels of proteins were determined based on densitometry analysis results. They are presented as mean ± standard deviation (SD) from three independent experiments. ** *p* < 0.01 vs. control, *** *p* < 0.001 vs. control.

Western blot was performed to investigate apoptotic protein expression levels in apigetrin-treated Hep3B cells. Results revealed that Bax, a pro-apoptotic protein, was increased by apigetrin in a dose-dependent manner. In contrast, the expression of Bcl-xl, an anti-apoptotic protein was decreased in apigetrin-treated cells (Figure 7). Discrepancy between pro-apoptotic and anti-apoptotic proteins favored apoptosis processes observed in apigetrin-treated cells. Furthermore, cleavage of caspase 3 and PARP occurred in treatment groups. These findings confirm that apigetrin treatment can induce apoptosis in Hep3B cells.

### 3.8. Effects of Apigetrin on Necroptotic Markers in Hep3B Cells

**Figure 8:**
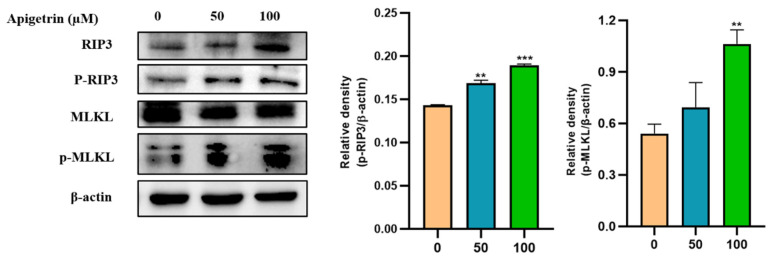
Effects of apigetrin on necroptotic markers. Treated Hep3B cells were harvested, and cell lysate was subjected to western blot. p-RIP3 and p-MLKL levels were calculated based on densitometry analysis. Results are expressed as mean ± standard deviation (SD) from three independent experiments. ** *p* < 0.01 vs. control, *** *p* < 0.001 vs. control.

Necroptosis is a regulated form of necrotic cell death. RIP3 and MLKL are crucial proteins that can trigger the necroptosis pathway. Immunoblot was used to examine protein expression levels of necroptotic markers on apigetrin-treated cells. It was observed that total and phosphorylation levels of RIP3 were up-regulated in cells treated with apigetrin. We also checked the expression levels of MLKL, which showed an increase in their phosphorylated form in treated cells, whereas the unmodified form of MLKL was unchanged (Figure 8).

## 4. Discussion

More than 60% of frequently used anticancer drugs originates from natural sources, including plants and marine species [28]. Natural products, including phytochemicals such as flavonoids, polysaccharides, lignans, alkaloids, terpenes, glycosides, saponins, and gums, are widely studied as effective anticancer agents [29]. Epidemiological studies have evaluated the significance of dietary flavonoids in reducing the risk of developing different cancers [21]. Apigetrin, also called cosmosin, is derived from *Scutellaria baicalensis Georgi*, *Teucrium gnaphalodes*, and *Taraxacum officinale* in fruits and vegetables [24,30]. In the current study, we elucidated the mechanism involved in the effect of apigetrin on Hep3B cells through apoptosis and necroptosis pathways.

MTT assay was performed to assess the viability of Hep3B cells after 24 and 48 h exposure to apigetrin at different concentrations. Cell viability was decreased by treatment with apigetrin in a concentration-dependent manner. Similarly, growth inhibitory effects of apigetrin-treated cells were analyzed by colony formation assay. Results showed decreased cell count and colony formation after apigetrin treatment, revealing the inhibitory effect of apigetrin on Hep3B cells. Previously, flavonoids have shown similar growth inhibitory effects of apigetrin on different cancer cells [31,32]. Cell cycle is complex process that involving multiple regulatory proteins that direct the cell through a specific sequence of events. Disruption of the G2/M checkpoint, also known as the DNA damage point, stops cell proliferation of damaged cells as this phase to maintain genomic stability [33]. Apigetrin caused a significant accumulation of Hep3B cells at the G2/M phase of the cell cycle, specifying cell cycle arrest. Likewise, previous reports have shown that apigetrin treatment can induce G2/M phase cell cycle arrest in AGS cells, supporting our current findings [34].

Apoptosis exhibits distinct morphological features such as condensation of chromosomes, fragmented nucleus, plasma membrane blebbing, cell shrinkage, and formation of apoptotic bodies [35]. DAPI staining was used to determine nuclear condensation with a blue fluorescent stain [36]. Necroptotic cells showed the visible formation of vacuoles, swelling of organelles, and loss of plasma integrity [37]. DAPI staining and hematoxylin staining showed similar morphological features. MTT assay, colony formation assay, and morphological observations revealed cell death, consistent with Annexin V/PI double staining results. Previous studies have found that berberine combined with cisplatin and oroxyloside can increase apoptotic and necroptotic populations in ovarian and hepatic cancer cells [38,39]. ROS can stimulate cell proliferation at lower levels while causing cell death at higher levels [40]. Flavonoids can increase ROS levels and stimulate apoptosis in cancer cells which can help in prevention and treatment of cancers [41]. ROS can act as a signaling molecule and a mediator of inflammation [42]. Our findings revealed that apigetrin could elevate ROS levels and contribute to cell death in Hep3B cells. Fluorescence microscopy revealed that NAC inhibited apigetrin-induced ROS production, and H_2_O_2_ was used as a positive control. Elevated ROS contributes to apoptosis and necroptosis cell death pathways [32,43].

TNFα is implicated in the pathogenesis of inflammatory and immune processes and malignancies by inducing mitochondrial ROS generation, contributing to cell death and initiation of necroptosis [42,44]. The NF-κB protein complex is considered as an apoptosis inhibitor and inducer of apoptosis through the NF-κB/RelA complex. This complex mediates apoptosis by actively downregulating NF-κB dependent, anti-apoptotic gene transcription [45]. Apigetrin treated Hep3B cells show inhibition of p-p65 and p-IκB phosphorylation. Similarly, fisetin treatment can result in NF-κB downregulation, leading to apoptosis of HT29 cells [46]. Increased TNFα can attenuate NF-κB activity in wogonin exposed leukemic cells and fisetin treatment in bladder cancer induces apoptosis through inhibition of NF-κB pathway [47,48]. Activation of Bax allows permeabilizing of the outer mitochondrial membrane to release cytochrome c into cytoplasm, where it binds to APAF-1 and triggers apoptosome formation [49]. In the present study, proapoptotic protein Bax was up-regulated, whereas anti-apoptotic Bcl-xl was down-regulated upon apigetrin treatment in Hep3B cells. Furthermore, cleaved caspase 3 and cleaved PARP were elevated by apigetrin, confirming the activation of the apoptotic pathway in Hep3B cells. Previously, kaemferol, fisetin, and apigenin phytochemicals have shown similar effects on apoptotic protein markers in cancer cell lines [19].

**Figure 9:**
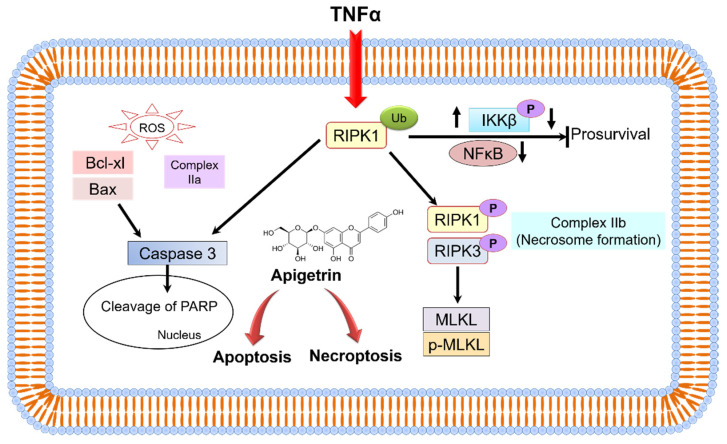
A schematic representation shows molecular mechanism involved in apigetrin-induced anticancer effects on Hep3B cells.

RIPK1-RIPK3 and mixed lineage kinase domain-like pseudokinase (MLKL) can activate the downstream of conventional death receptor-mediated necroptotic mechanisms [50]. TNFα-induced necroptosis is mediated by RIPK1 and RIPK3 markers. Results obtained from double staining, morphological observations, and immunoblotting of necroptosis marker proteins RIPK3, MLKL, p-RIPK3, and p-MLKL showed induction of necroptosis in apigetrin-treated Hep3B cells. Previously studied compounds such as fisetin and quercetin induced apoptosis and necroptosis in cancer cells [51,52]. The present study demonstrates that apigetrin can induce necroptosis and apoptosis by inhibiting the NF-κB signaling pathway in Hep3B cells (Figure 9).

## 5. Conclusions

In summary, findings of this study show that flavonoid apigetrin (Apigenin-7-glucoside) can inhibit the proliferation of human HCC, Hep3B cells. Apigetrin exhibited anticancer potential through necroptosis and apoptosis cell death pathways. Our findings provide a strong basis for future research on apigetrin as a possible anticancer agent for treating hepatocellular cancer. More experimental trials, particularly in vivo models, are required as future validations studies to prove apigetrin’s anticancer potential via apoptotic and necroptotic mechanisms.

## Data Availability

The data used to support the findings of this study are available from the corresponding author upon reasonable request.
